# Determination of barbiturates in hair samples by using a validated UHPLC-HRMS method: application in investigation of drug-facilitated sexual assault

**DOI:** 10.1080/20961790.2019.1659474

**Published:** 2019-10-18

**Authors:** Di Wen, Yan Shi, Xiaoguang Zhang, Bing Xie, Wenqiao Liu, Feng Yu, Ping Xiang, Bin Cong, Chunling Ma

**Affiliations:** a College of Forensic Medicine, Hebei Medical University, Hebei Key Laboratory of Forensic Medicine, Collaborative Innovation Center of Forensic Medical Molecular Identification, Shijiazhuang, China; b Academy of Forensic Science, Ministry of Justice, Shanghai Key Laboratory of Forensic Medicine, Shanghai, China; c Core Facility of Hebei Medical University, Shijiazhuang, China

**Keywords:** Forensic sciences, forensic toxicology, segmental hair analysis, barbiturates, UHPLC-HRMS, drug-facilitated sexual assault

## Abstract

In recent years, benzodiazepines and benzodiazepine-like drugs are the most common substances associated with drug-facilitated sexual assaults (DFSA); however, barbiturates are also detected occasionally. Segmental hair analysis provides useful information on the historic pattern of drug use, enabling differentiation between single exposure in DFSA cases and chronic use. However, sensitive and specific methods for barbiturate analysis in hair samples are needed. Herein, we present an ultra-high-performance liquid chromatography coupled with high-resolution mass spectrometry (UHPLC-HRMS) method for qualitative and quantitative determination of seven barbiturates in hair samples. Firstly, a hair strand was decontaminated and then freeze-milled in liquid nitrogen. Next, 50 mg of powdered hair was extracted with methanol in an ultrasonic bath for 10 min in the presence of 10 ng phenobarbital-d5. The supernatant was dried under nitrogen gas, and the pellet was dissolved in 100 µL mobile phase. Afterwards, 10 µL of the suspension was injected into the UHPLC-HRMS system. The present method involved two UHPLC conditions for determination of barbiturates (I) and identification of the structural isomers amobarbital and pentobarbital (II). This method showed satisfactory linearity in a range of 0.02–20.00 ng/mg for UHPLC conditions I and II, both with a high determination coefficient (0.9991–0.9999). The selectivity, intra- and interday precision, accuracy and matrix effect of the method were acceptable. Next, the validated method was applied to investigate an authentic DFSA case. Hair samples (black, approximate 25 cm long) were collected 3 months after the assault, and the proximal segments (0–5 cm from the root; each segment was 1 cm long) were analyzed. Amobarbital was detected at a concentration of < LOQ (limit of quantification) and 0.09 ng/mg in the second and third 1 cm hair segment but not in the other segments. Thus, our method was successful in determining barbiturate concentration in human hair after a single-dose exposure, showing its potential for application in the investigation of DFSA cases.

## Introduction

Recently, drug-facilitated sexual assaults (DFSA) are increasingly severe in China [1,2]. However, drug determination is a challenge for forensic toxicologists in DFSA cases due to the target drug concentrations in blood and urine are mostly very low and difficult to detect when the assault was reported. The development of analytical techniques has enabled sensitive determination of drugs in unconventional matrices, such as hair [3–5]. Hair is a unique biological sample for retrospective detection of drug exposure when the sampling procedure in DFSA case was delay [6–9]. Segmental analysis of hair strands provides useful information on historic pattern of drug use, enabling differentiation between single-dose exposure in DFSA cases and chronic use in clinic therapy [10–13]. For these reasons, hair is an excellent biological sample with particular value in DFSA cases [14,15].

A series of literature [14,16–18] showed several DFSA cases in China and other countries, with benzodiazepines and benzodiazepine-like drugs as the most common substances associated with DFSA. Barbiturates, with their addictive characteristics and serious side effects, were replaced by benzodiazepines in clinical practice [19]. Nevertheless, even today, some barbiturates such as phenobarbital are still being prescribed for therapy of epilepsy seizures, especially in developing countries owing to the low cost of these drugs. In addition, poisoning cases caused by barbiturates occur occasionally [20]. Goldblum et al. [21] in 1954 firstly reported that phenobarbital, after administration, enters the hair and is deposited there for 6 or more days. Different extraction techniques and analytical methods have been built to determine multiple drugs in hair samples, especially those based on solid-phase extraction and gas/liquid chromatography coupled with mass spectrometry (GC/LC-MS) [22–25]. However, very few methods have been conducted in DFSA cases to determine barbiturates in hair samples because the level of barbiturates incorporated into the hair is low and the sensitivity of the method is not always adequate in cases of single-drug exposure. High-resolution mass spectrometry (HRMS) [26] and time-of-flight mass spectrometry (TOF-MS) [27] offer the advantage of accurate mass measurement and good sensitivity; thus, their uses for drug determination in biological specimens are becoming increasingly common. Therefore, it is important to develop and validate a method for determination of barbiturates in hair samples for application in DFSA cases.

In this study, we present an ultra-high-performance liquid chromatography coupled with high-resolution mass spectrometry (UHPLC-HRMS) method that allowed qualitative and quantitative determination of seven barbiturates, as well as identification of the structural isomers amobarbital and pentobarbital in hair samples after freeze-milling and methanol extraction. Furthermore, this method was fully validated and applied to analyze an authentic hair sample from a DFSA case.

## Materials and methods

### Standards and reagents

Barbital, phenobarbital, amobarbital, pentobarbital, secobarbital, butalbital, thiopental sodium standard and the internal standard (IS) phenobarbital-d5 were purchased from Cerilliant Corporation (Round Rock, TX, USA). HPLC grade of acetonitrile, acetone, methanol and ammonium acetate were the productions of Fisher Scientific (Fair Lawn, NJ, USA). Ultrapure water was produced in the laboratory using a Milli-Q system (Millipore, Billerica, MA, USA).

### Sample preparation

The blank hair samples used for quality control (QC) and validation of the method were obtained from healthy volunteers with no history of barbiturate consumption. All samples were collected with each volunteer’s consent.

The hair samples were prepared using the following method. Firstly, to remove undesired contaminants that may cause interferences with the analysis from the hair surface, the hair samples were rinsed once with 1% detergent, twice with pure water, and once with 5 mL acetone. The acetone wash solution was dried under nitrogen gas and the pellet was stored for the further check of external contamination. After external decontamination, the washed hair was dried at room temperature and subsequently divided into 1 cm-long segments. The hair segments were cut into small pieces of shorter than 2 mm and then delivered to a liquid nitrogen milling apparatus (6775 Freezer/Mill^®^; SPEX SamplePrep, Metuchen, NJ, USA) for pulverization. Next, 50 mg of powdered hair was extracted with 5 mL methanol in a tube, and then sonicated in an ultrasonic bath (40 kHz) for 10 min in the presence of 10 ng IS. The tube was centrifuged for 5 min at 12 000 r/min and 4 °C. Next, the supernatant was filtered using a 0.2 µm membrane and dried under nitrogen gas. Finally, the pellets were dissolved in 100 µL mobile phase (80% 10 mmol/L ammonium acetate and 20% acetonitrile), and 10 µL of the suspension was injected into the chromatographic system.

### UHPLC-HRMS conditions

Thermo Scientific™ Q Exactive™ Focus (Thermo Scientific, Inc., USA), a UHPLC-HRMS system, was utilized for automated screening, profiling and quantification analyses for this study. The present method involved two different UHPLC conditions for determination of barbiturates (I) and separation of structural isomers (II).

#### UHPLC condition I

The column used for determination of barbiturates in this study was a Thermo Scientific Hypersil GOLD^TM^ C-18 column (100 × 2.1 µm, 1.9 µm; Thermo Scientific, Inc.) protected by the matched Security Guard C-18 pre-columns. The gradient elution was as follows: 0.0–8.0 min: phase A from 95% to 5% and phase B from 5% to 95%; 8.01–10.0 min: 5% phase A and 95% phase B; 10.01–12.0 min: 95% phase A and 5% phase B. Mobile phase A was 10 mmol/L ammonium acetate, and mobile phase B was acetonitrile. The flow rate was 300 µL/min.

#### UHPLC condition II

We optimized the UHPLC condition to separate the structural isomers amobarbital and pentobarbital. Thermo Scientific Hypersil GOLD^TM^ C-18 column (100 × 2.1 µm, 1.9 µm; Thermo Scientific, Inc.), Phenomenex Kinetex^®^ EVO C-18 column (100 × 2.1 µm, 2.6 µm; Phenomenex Inc., USA) and Waters ACQUITY^®^ BEH C-18 column (100 × 2.1 µm, 1.7 µm; Waters Inc., Dublin, Ireland) were used and protected by the matched Security Guard C-18 pre-columns. Three UHPLC elution conditions were investigated, including the gradient elution procedure used in UHPLC condition I and two isocratic elution procedures at a flow rate of 200 µL/min performed under 80% using 10 mmol/L ammonium acetate or pure water and 20% acetonitrile.

The optimal HRMS parameters selected were as follows: spray voltage, 3200 V; capillary temperature, 320 °C; aux gas heater temperature, 300 °C; sheath gas (nitrogen) rate, 30 L/min; aux gas (nitrogen) rate 15 L/min. The optimal multiple reaction for monitoring transitions and respective collision energy (CE) for precursor and secondary ions of barbiturates and IS were determined by consecutive injections of the individual standards at a concentration of 100 ng/mL and analyzed by the software of Thermo Scientific Q Exactive (Thermo Scientific, Inc.). The optimal HRMS parameters selected were used for both UHPLC conditions.

### Validation of the assay

#### Selectivity

Validation of the assay was performed according to the guideline raised by Peters et al. [28]. Specific MS_1_ extracted chromatograms were assessed by analyzing nine different blank hair samples for evaluating selectivity of UHPLC-HRMS analysis. The acceptance criterion of selectivity was no interfering peaks at retention times of analytes.

#### Linearity and limit of detection and quantification

Mixed standard working solutions of barbiturates (diluted with methanol) were spiked to blank hair to get a series of standard concentrations at 0.02, 0.05, 0.1, 0.2, 0.5, 1, 10 and 20 ng/mg. The linearity of the method for determination of barbiturates was studied in the range of limit of quantification (LOQ) to 20 ng/mg, with triplicate analyses for each level. Calibration curves were generated through linear regression of the peak area ratio under the specific extracted precursor ion (MS_1_) chromatograms of barbiturates versus IS with the concentration of barbiturates. The limit of detection (LOD) was the concentration that had a signal-to-noise ratio of >3%, and the LOQ was considered as the lowest point of the calibration curve that could be determined with signal-to-noise ratio >10% and ion ratios equal to or lower than 20% deviation.

#### Accuracy and precision

QC samples were prepared by adding stock solution of barbiturates to blank hair samples, and the final concentrations of barbiturates were 0.02, 0.04 and 0.2 ng/mg. Accuracy was evaluated as percentage deviation of the mean from the true value. Precision was expressed as relative standard deviation (RSD) at each QC level. Intraday precision was evaluated by six replicates of each QC level in 1 day, and interday precision was assessed by the analysis on 3 days.

#### Extraction recovery and matrix effect (ME)

Extraction recovery was established, at three QC levels, by comparing six replicates of the analyte peak areas of extracted spiked samples with those of blank hair samples spiked with the same amounts of the barbiturate after extraction. ME was quantitatively assessed by comparing the slope of calibration curves [29] (LOQ: 20 ng/mg) obtained from barbiturate post-spiking samples (*k*) to those from neat calibration solution (*k*_0_) at different barbiturate concentrations, and defined as ME% = (*k*/*k*_0_ − 1) × 100.

### Method application

The present method was applicated in an authentic DFSA case. The victim was a 23-year-old woman; she drank a bottle of juice when having dinner with a male friend, and then felt lethargic and dizzy. No drugs were detected in her blood sample, which was collected several days after she was raped by that male. Hence, her hair sample was collected 3 months later. She had no recent use of medication or illicit substances. The hair sample, approximately 25 cm long, was cut directly above the skin at the back of the head, and then stored under dry conditions at room temperature. The hair color was black, and the proximal segment (0–5 cm from the root) was analyzed. Hair sample collection was conducted following the general guidelines [9,30].

## Results

### UHPLC-HRMS conditions

**Figure 1.**
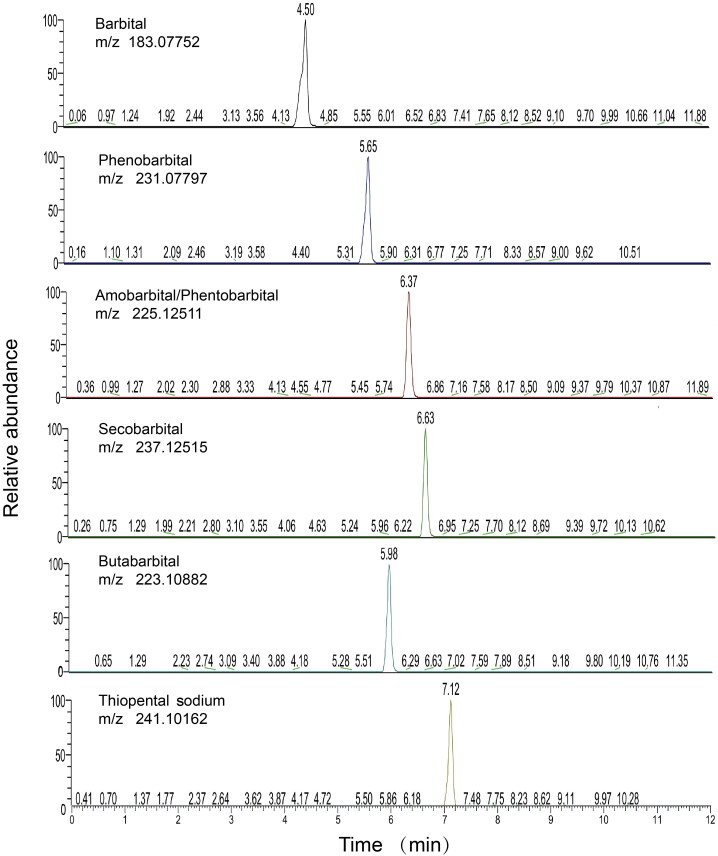
Extracted ionic chromatogram obtained from UHPLC-HRMS analysis of 0.2 ng/mg barbiturates in spiked blank hair samples. A mixture of barbital, phenobarbital, amobarbital, pentobarbital, secobarbital, butalbital and thiopental sodium (10 ng each) was spiked into 50 mg of blank hair sample.

The extracted ion chromatograms (Figure 1) recorded on a Thermo Scientific Hypersil GOLD^TM^ C-18 column under UHPLC condition I from a blank hair sample spiked with all analytes at 0.2 ng/mg for barbiturates and 0.5 ng/mg for IS were acquired. The run time was 12.00 min, and all compounds were eluted between 4.50 and 7.12 min; no significant interference was found in the detection of barbital, phenobarbital, secobarbital, butalbital, thiopental sodium and IS. However, amobarbital and pentobarbital could not be separated under UHPLC condition I.

**Figure 2.**
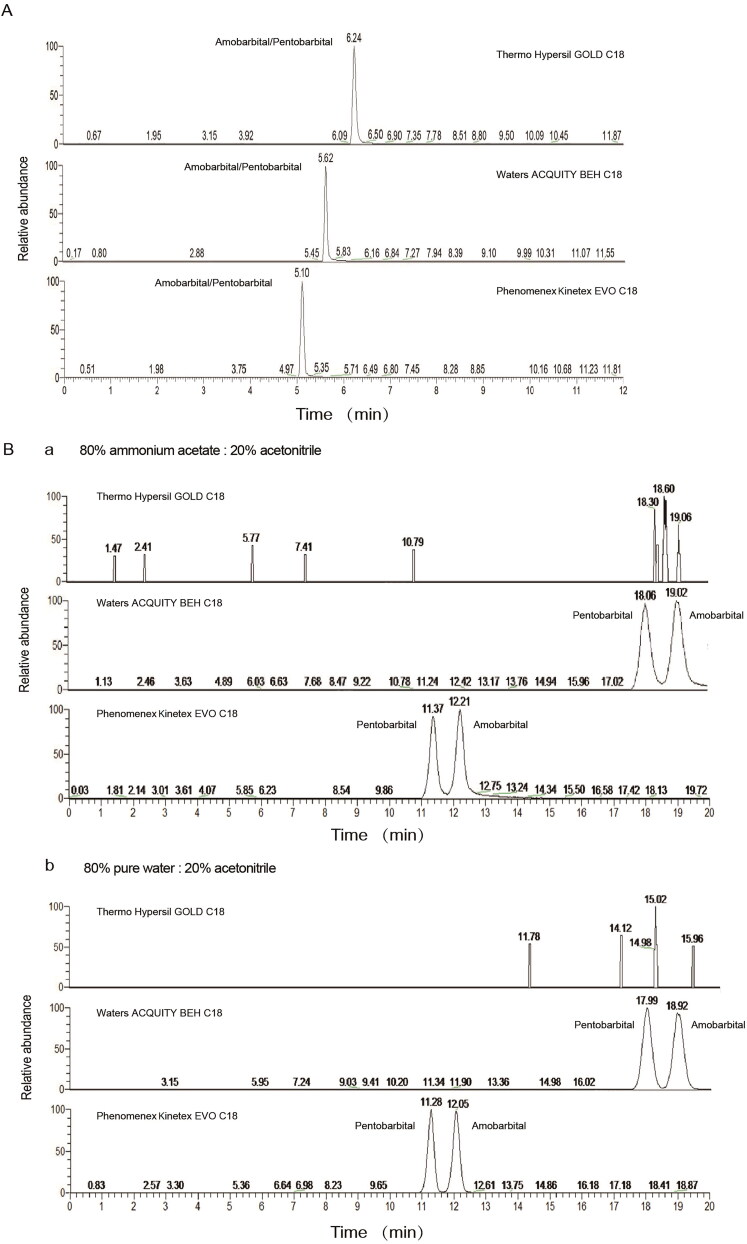
Extracted ionic chromatogram obtained from UHPLC-HRMS analysis of 0.2 ng/mg amobarbital and pentobarbital in spiked blank hair samples. A mixture of amobarbital and pentobarbital (10 ng each) was spiked into 50 mg of blank hair sample. The efficiency of gradient elution (A) and isocratic elution (B) was evaluated on Thermo Hypersil GOLD^TM^ C-18 column, Waters ACQUITY^®^ BEH C-18 column and Phenomenex Kinetex^®^ EVO column. The mobile phases for isocratic elution were 80% 10 mmol/L ammonium acetate (B-a) or pure water (B-b) and 20% acetonitrile.

As shown in Figure 2(A), three columns were non-effective to separate amobarbital and pentobarbital under UHPLC condition I. However, Phenomenex Kinetex^®^ EVO C-18 column and Waters ACQUITY BEH^TM^ C-18 column with isocratic elution under UHPLC condition II successfully separated amobarbital and pentobarbital (Figure 2(B)). Notably, isocratic elution with 80% pure water and 20% acetonitrile on Phenomenex Kinetex^®^ EVO C-18 column showed the highest efficiency in separating the structural isomers amobarbital and pentobarbital.

### Validation of the assay

#### Selectivity

The selectivity was confirmed by the absence of interfering peaks at the retention times for barbiturates in blank hair powders. The retention times, precursor ion (MS_1_) and secondary ions (MS_2_) of barbiturates and the IS phenobarbital-d5 were showed in Table 1.

**Table 1.** UHPLC-HRMS parameters for barbiturate analysis.

	Compound	Chemical formula	Retention time (min)	Ionization form	MS_1_ (*m/z*)	MS_2_ (*m/z*)
UHPLC condition I	Barbital	C_18_H_12_N_2_O_3_	4.50	[M-H]	183.07752	140.07207 85.00452
	Phenobarbital	C_12_H_12_N_2_O_3_	5.65	[M-H]	231.07797	188.07214 85.00438
	Amobarbital/Pentobabital	C_11_H_18_N_2_O_3_	6.37	[M-H]	225.12511	182.11923 85.00434
	Secobarbital	C_12_H_18_N_2_O_3_	6.63	[M-H]	237.12515	194.11917 85.00447
	Butalbital	C_11_H_16_N_2_O_3_	5.98	[M-H]	223.10882	180.10294 85.00420
	Thiopental sodium	C_11_H_17_NaN_2_O_3_	7.12	[M-Na]	241.10162	57.97557 100.98112
	Phenobarbita-d5	C_12_H_17_N_2_O_3_	5.65	[M-H]	236.07728	193.10257 85.00412
UHPLC condition II	Pentobabital	C_11_H_18_N_2_O_3_	11.28	[M-H]	225.12511	182.11923 85.00434
	Amobarbital	C_11_H_18_N_2_O_3_	12.05	[M-H]	225.12511	182.11923 85.00434
	Phenobarbita-d5	C_12_H_17_N_2_O_3_	4.60	[M-H]	236.07728	193.10257 85.00412

#### Linearity, LOD and LOQ

As shown in Table 2, the method showed broad linearity of LOQ to 20 ng/mg for barbital, phenobarbital, amobarbital, pentobarbital, secobarbital, butalbital and thiopental sodium, with correlation coefficients (*r*^2^) > 0.999 for all analytes under UHPLC condition I. LOQ corresponded to the first calibration point, which was 0.02 ng/mg. LODs of each barbiturate under UHPLC condition I are 0.01 ng/mg. In addition, the linearity, LOD and LOQ of the method under UHPLC condition II for determination of amobarbital and pentobarbital at the same time are also presented in Table 2.

**Table 2.** Linearity, limit of detection (LOD) and limit of quantification (LOQ) of barbiturates.

	Compound	Linearity	*r* ^2^	LOQ (ng/mg)	LOD (ng/mg)
UHPLC condition I	Barbital	*y* = 0.007856*x* − 0.005332	0.9997	0.02	0.01
	Phenobarbital	*y* = 0.010555*x* − 0.01244	0.9998	0.02	0.01
	Amobarbital	*y* = 0.020249*x* + 0.023212	0.9991	0.02	0.01
	Pentobarbital	*y* = 0.00801162*x* − 0.003577	0.9991	0.02	0.01
	Secobarbital	*y* = 0.010223*x* − 0.019752	0.9993	0.02	0.01
	Butalbital	*y* = 0.011896*x* − 0.026402	0.9999	0.02	0.01
	Thiopental sodium	*y* = 0.008356*x* − 0.043479	0.9995	0.02	0.01
UHPLC condition II	Amobarbital	*y* = 0.00841701*x* − 0.01249	0.9999	0.02	0.01
	Pentobarbital	*y* = 0.00875395*x* + 0.00236192	0.9998	0.02	0.01

#### Recovery of extraction and ME

Table 3 presents the average values obtained for average recovery with the RSD% obtained by using different hair samples and different concentrations of analytes. The recovery ranged from 91.0% to 119.7%. Table 3 shows the mean repeatability and accuracy results obtained at three different concentrations, and the method presented satisfying intra- and interday precision and accuracy. Furthermore, the calculated ME ranged from −1.29% to −9.05%, showing no obvious signal enhancement or inhibition of the proposed analytical procedure.

**Table 3.** Recovery, intra- and interday precision and matrix effect (ME) for barbiturates.

	Compound	Concentration (ng/mg)	Average recovery (%)	CV (%)	Precision (CV, %)		ME (%)
					Intraday (*n* = 6)	Interday (*n* = 3)	
UHPLC condition I	Barbital	0.02	101.9	2.38	2.06	2.72	−1.29
		0.04	91.0	5.10	2.30	3.80	
		0.20	99.2	4.96	1.36	4.56	
	Phenobarbital	0.02	108.0	1.90	1.24	2.46	−4.12
		0.04	99.3	1.14	0.77	1.29	
		0.20	99.8	0.27	1.20	2.46	
	Amobarbital	0.02	95.5	5.57	3.39	2.44	−2.83
		0.04	102.8	1.90	3.39	1.97	
		0.20	92.6	2.46	2.33	3.16	
	Pentobarbital	0.02	95.6	5.31	1.01	4.00	−2.67
		0.04	99.7	4.61	1.89	0.99	
		0.20	101.5	1.03	1.24	1.02	
	Secobarbital	0.02	117.2	2.21	1.36	3.58	−8.43
		0.04	101.4	2.90	2.56	4.71	
		0.20	103.4	3.34	4.75	3.54	
	Butalbital	0.02	119.7	2.52	3.42	1.62	−9.05
		0.04	101.3	4.70	2.28	2.02	
		0.20	101.3	1.98	2.24	6.07	
	Thiopental sodium	0.02	93.7	3.82	3.73	5.21	−4.61
		0.04	106.8	3.91	5.86	1.96	
		0.20	107.1	1.69	4.89	2.20	
UHPLC condition II	Amobarbital	0.02	101.8	3.44	3.01	1.78	−2.21
		0.04	98.6	2.08	3.46	4.41	
		0.20	99.7	1.56	1.05	2.87	
	Pentobarbital	0.02	98.5	2.17	2.89	3.09	−3.36
		0.04	96.6	3.39	2.60	2.54	
		0.20	102.1	1.65	1.09	2.90	

CV: coefficient of variance.

### Method application

**Figure 3.**
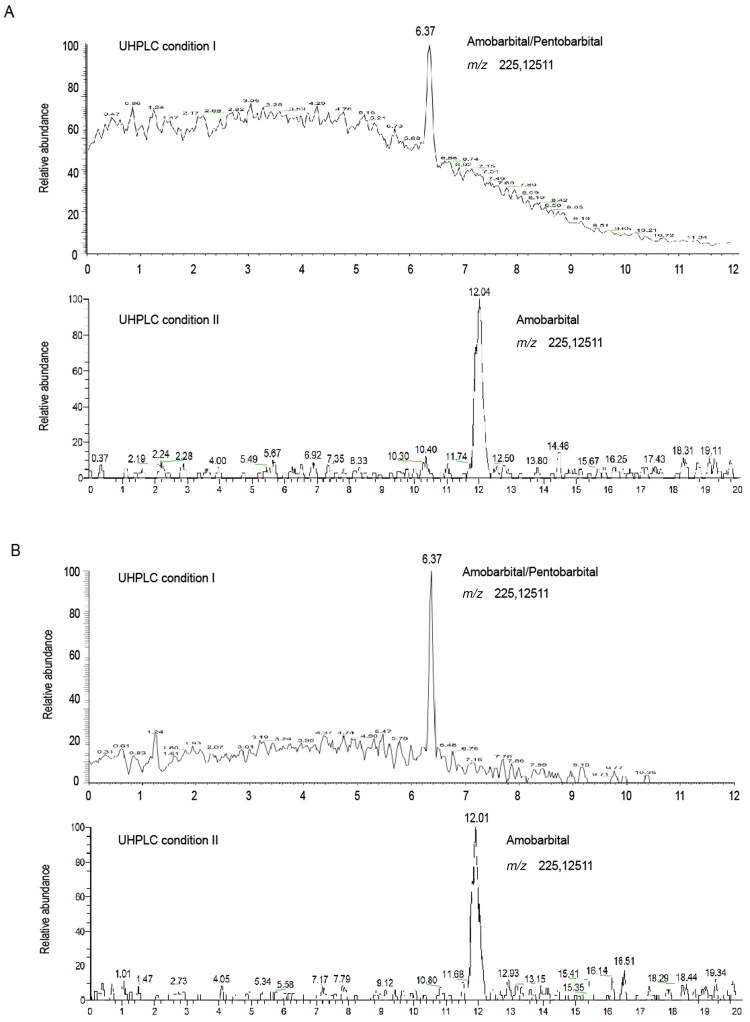
Extracted ionic chromatogram obtained from the optimized UHPLC-HRMS analysis of amobarbital in authentic hair samples (A: 1–2 cm segment; B: 2–3 cm segment).

The method was applied to an authentic sample from a DFSA case. Toxicological analysis was performed on the blood sample isolated several days following the assault. Regrettably, the results indicated no drug in the blood sample, whereas urine sample was not collected and, thus, unavailable for forensic toxicological analysis. The male suspect did not confess to having given the victim any drugs and denied charges of rape. Consequently, a hair sample was collected from the back of the victim’s head 3 months later. The hair was decontaminated and cut into 1 cm-long segments. The results of analysis by the present method showed that amobarbital was detected in the second- and third-centimetre segment of the hair strand (Figure 3) but not detected in the other segments. Amobarbital content in the second- and third-centimetre segments was < LOQ and 0.09 ng/mg, respectively.

## Discussion

The number of DFSA has dramatically increased over the last few years [31,32]. Benzodiazepines and benzodiazepine-like “Z” compounds, such as clonazepam, estazolam, midazolam and zolpidem are usually reported in DFSA cases [17,33]. In contrast, barbiturate findings in DFSA cases are currently rare, and little is known regarding the concentration of barbiturates in the hair. Herein, we developed and validated a simple, low LOD, and reliable procedure to determine barbiturates in hair samples based on liquid nitrogen milling, methanol extraction and UHPLC-HRMS analysis.

For barbiturate analysis of hair samples, the sample preparation procedure should include toxicologically relevant acidic substances with high extraction yield and should avoid hydrolysis or decomposition [7,34]. Freeze milling in liquid nitrogen environment was selected because it allowed pulverization of each hair segment separately in milling tubes and reduced the risk of contamination between samples. Furthermore, compared to hair digestion, it allowed the complete release of compounds and no analyte degradation. The subsequent extraction method was performed using methanol, a non-reactive and universal organic solvent, in an ultrasonic bath. The results showed that the sample preparation procedure had a high extraction efficiency.

Next, we validated the barbiturate determination assay of hair samples using the UHPLC-HRMS system. The sensitivity of the method is of crucial importance. Montesano et al. [35] reported a method for targeted analysis of 96 drugs, including phenobarbital and thiopental, in hair samples by UHPLC-MS/MS. The LOD of that method for phenobarbital and thiopental determination was 1 and 0.1 ng/mg, respectively. Compared with the previous method, the method presented here was more sensitive for determining seven barbiturates and showed a lower LOD of approximately 0.01 ng/mg. Moreover, the validation results indicated that our method had good linearity, accuracy and precision, with no obvious signal enhancement or inhibition of barbiturates in hair matrices. However, it is noteworthy that pentobarbital, a structural isomer of amobarbital, could not be separated under UHPLC condition I. Nevertheless, pentobarbital is a metabolite of thiopental, and both compounds were detected in the hair of a woman who was hospitalized after being sexually assaulted [20]. To identity pentobarbital and amobarbital, we designed another UHPLC condition with Phenomenex Kinetex^®^ EVO column and isocratic elution procedure, allowing simultaneous determination of amobarbital and pentobarbital.

The authentic hair sample was cut into five 1 cm-long segments, which, assuming a hair growth rate of 1 cm/month [36], corresponded to approximately the previous 5 months. External contamination was firstly examined by analyzing the last acetone wash. In the third segment, which corresponded to the time of the crime, amobarbital was detected at a concentration of 0.09 ng/mg. Interestingly, amobarbital was detected at a low level (<LOQ) in the second segment. Most studies show no abrupt change from a positive to a negative result within a sample corresponding to the stop of drug intake. Shen et al. [37] summarized several possible reasons for this phenomenon. We assumed that considerable variabilities in the isolation of hair from the scalp and in the sectioning of hair samples were the main factors underlying this result. Kintz et al. [38] determined the concentrations of phenobarbital (21.7–137.3 ng/mg), amobarbital (31.4–41.6 ng/mg) and secobarbital (21.6–58.9 ng/mg) in hair samples collected from chronic drugs users, including those collected post-mortem. The concentration of amobarbital in our study was much lower than that in these previous studies and indicated a single-dose exposure to the drug in this particular DFSA case. Moreover, the victim had no recent use of medication or illicit substances; no benzodiazepines and benzodiazepine-like drugs were detected in each segment of the hair strand by a routine UHPLC-HRMS method in our laboratory. At last, the criminal suspect admitted the fact that he bought the unknown drugs online and administered to the victim.

Hair pigmentation appears to be an important factor in drug incorporation. The binding mechanism of a drug to melanin pigments has been clarified by several studies [39,40]. Compared to light hair, darker hair contains more melanin, which leads to a greater accumulation of drugs. However, it is generally acknowledged that cationic charge of drug molecules is important in determining their incorporation into the hair and their binding to melanin; in contrast, hair incorporation of anionic or neutral drugs, such as phenobarbital, would not be influenced by pigmentation. There is limited literature on the disposition of barbiturates in biological matrices other than the blood and urine, and to our knowledge, this is the first study of amobarbital concentration in the hair. Thus, we could not make direct comparisons to other similar publications. Controlled studies of a single-dose use in humans, e.g., the study on ketamine by Xiang et al. [41], will be performed to advance our understanding of barbiturate levels in the hair.

In conclusion, a fast and sensitive UHPLC-HRMS method was validated for determination of seven barbiturates in hair samples, and the method was successful in determining barbiturate concentration after a single-dose exposure in an authentic human hair sample from a DFSA case.

## Authors’ contributions

All authors contributed to the writing of the manuscript and approved the final version.
